# Deployment of a herd health package increases production traits and reduces parasite load and disease incidence in sheep and goats in Mali

**DOI:** 10.3389/fvets.2026.1894736

**Published:** 2026-08-07

**Authors:** Olivier Mahuton Zannou, Cheick Amadou Tidiani Diakité, Chaka Traoré, Ahmadou Sow, Ons Tebourbi, Mourad Rekik, Michel Dione

**Affiliations:** 1 International Livestock Research Institute, Bamako, Mali; 2 Laboratoire Central Vétérinaire, Bamako, Mali; 3 International Center for Agricultural Research in the Dry Areas, Tunis, Tunisia

**Keywords:** deworming, gastrointestinal parasites, herd health, Mali, small ruminants, vaccination

## Abstract

Small ruminant production plays a crucial role in rural livelihoods and food security in Mali but is constrained by infectious diseases, parasitic infections, poor nutrition, and limited preventive healthcare. This study evaluated the effect of implementing a herd health package on the productivity, parasite load, and disease incidence in small ruminant herds in the Farakala commune, Sikasso region, Mali. This package included regular, targeted deworming campaigns, vaccination against Peste des Petits Ruminants, and training for livestock farmers on best practices regarding feeding, hygiene, housing, and reproduction in small ruminants. A total of 120 growing sheep and goats (2–6 months old) were monitoredfor 1 year (October 2023–September 2024) over 24 herds divided into test (with package) and control (without package) groups. Data on weight gain, herd growth rate, reproductive performance, and occurrence of gastrointestinal parasites were monitored. We used the linear model and the generalized linear model to check if response variables had a significant effect on productivity, parasite load, and disease incidence in small ruminant herds. We also used Mixed Models to check the impact of the package on small ruminant herds after its implementation. Results showed that Test herds experienced significantly fewer health conditions (OR = 0.62; *p* < 0.05) and higher herd growth rates (*β* = 23.7%; *p* < 0.05) than Control herds. Average daily weight gain was greater in treated herds (*β* = 8.8 g/day; *p* < 0.05), and live births were 1.65 times higher compared to controls (*p* < 0.05). Coprological analyses revealed four main gastrointestinal parasite genera (Strongylid nematodes, *Eimeria* spp., *Trichuris* spp., and *Moniezia* spp.), with significantly lower incidence in treated herds (OR = 0.43; *p* < 0.05). Sheep were more heavily infected than goats before (OR = 2.14; *p* < 0.05) and after intervention (OR = 1.49; *p* < 0.05). The integrated herd health package effectively improved small ruminant productivity by enhancing growth while reducing parasitic load and disease incidence. Scaling up this approach, combined with continuous farmer training and monitoring, could substantially improve livestock performance and rural incomes in similar production systems.

## 1 Introduction

Farming of small ruminants (sheep and goats) makes an important contribution to food security in Mali through the production of meat and milk. It also makes a considerable contribution to the income of rural populations, especially women in the case of small ruminants (1). Unfortunately, this type of livestock farming faces many challenges, including a high burden of animal diseases and poor management practices, leading to reduced herd productivity and profitability. Among these health challenges, gastrointestinal parasites (GIPs) have a negative impact on small ruminant production through direct and indirect losses. Direct losses consist of mortalities, precocious slaughter, and meat depreciation during veterinary inspection at the slaughterhouse. Indirect losses are represented by reduced productivity as a consequence of opportunistic diseases (2). Animal infection by GIPs is closely linked to the type of farming method practiced. Over 70% of ruminants in the West African sub-region are raised under a pastoralist system. This type of farming method exposes animals to parasite infection, especially when the pastures are grazed by several herds at the same time. In addition, the scarcity of pastures in some areas due to periodic drought leads to overgrazing, which makes these pastures heavily infested (3).

Another health challenge for small ruminant farming is Peste des Petits Ruminants (PPR) or “goat plague.” PPR is a highly contagious viral disease that serves as a major health constraint, causing up to 90% mortality in sheep and goat flocks across Africa, Asia, and the Middle East. It causes severe economic losses, destroying livelihoods, threatening food security, and severely limiting trade due to high sickness and death rates (4, 5). However, poor management practices, such as lack of vaccination, uncontrolled animal movement, crowding, and poor hygiene, are primary drivers for its spread, persistence, and high mortality rates in small ruminant populations (6, 7). While PPR is a severe threat, it is considered a priority disease for control and eventual eradication through consistent vaccination campaigns and improved veterinary care (8).

Traditional animal health systems in many sub-Saharan African countries often rely on reactive therapeutic interventions that address individual animals only after symptoms appear. This approach is increasingly viewed as insufficient for achieving sustainable gains in animal welfare and farm profitability (9). Consequently, there is a growing shift toward herd health management, a proactive, integrated approach that combines preventive veterinary care (such as strategic vaccinations and deworming) with good management practices in nutrition and husbandry (10).

Building on the hypothesis that integrating health with improved management can overcome the limitations posed by treatments of individual animals, this study, a randomized controlled trial, evaluates the deployment of a comprehensive herd health package in Mali. We assessed its impact by (i) measuring gains in growth and weight; (ii) quantifying the reduction in gastrointestinal nematode loads; (iii) monitoring health issues.

By analyzing these key indicators, we aim to demonstrate how a systemic herd health package can serve as a scalable solution for strengthening weak animal health systems in similar resource-limited environments.

This paper reports the results of an evaluation carried out from October 2023 to September 2024 in the Farakala commune, an agro-pastoral setting in central Mali, to investigate the effect of a herd health package on the selected small ruminant herds.

## 2 Materials and methods

### 2.1 Study area

**Figure 1:**
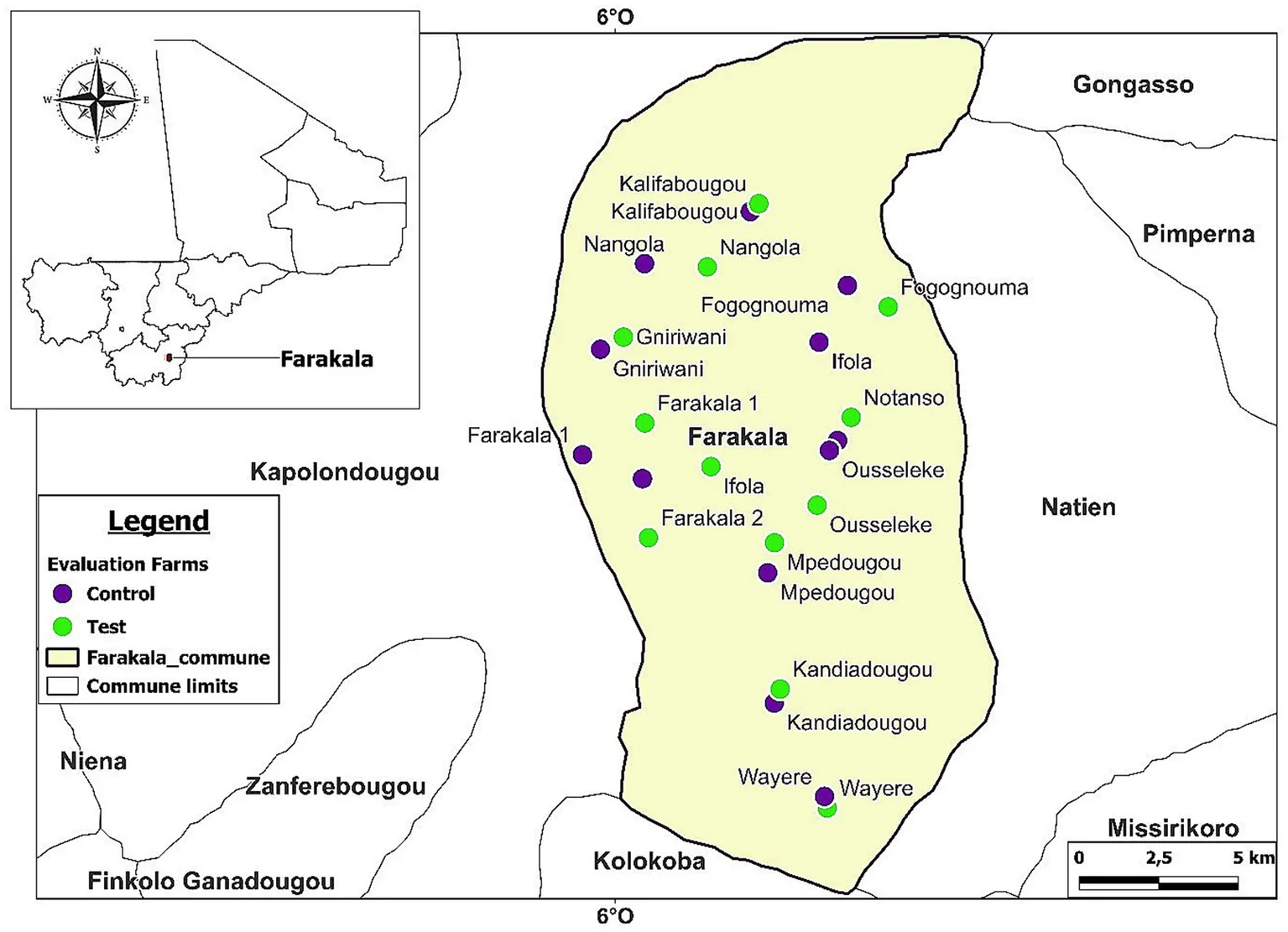
Study area with details on geographical distribution of selected herds in the commune of Farakala. In the upper-left corner of the map, in a medallion, is the location of the town of Farakala on the map of Mali.

The present study took place in the commune of Farakala in the western part of the Sikasso Cercle in the Sikasso Region, central Mali. (Figure 1). This commune is made up of 12 villages: Farakala 1, Farakala 2, Fogognouma Diassa, Kalifabougou, Gniriwani, Nangola, Kandiadougou, Ousseleke Diassa, Ifola, Notanso, Mpedougou, and Wayere. It is crossed by the national road N°7, which passes through Mpedougou and Farakala. The study took place in commune of Farakala. In the study area, the small ruminant production system is semi-intensive agro-pastoral with limited seasonal transhumance. Feed resources are made of mainly crop residues during dry season and pastures during wet season. Veterinary services are limited with most farmers facing difficulty in accessing vaccines and treatments.

### 2.2 Study design and population

This study was carried out from October 2023 to September 2024, in Farakala, an agro-pastoral commune in central Mali, to investigate the effect of a herd health package on small ruminant herds. The farmers who participated in this study were selected in each of the 12 villages of the commune through a random draw from the list of volunteers who expressed a willingness to take part in the experiment. The assignment of herd type (Test or Control) was also determined by random draw during public meetings with the farmers. All the animals of a Test farm received the herd health package whereas the Control herds animals did not receive the package during the study but at the end.

The target animal population consisted of sheep and goats reared in the commune of Farakala. We selected only growing animals aged 2–6 months. This choice was justified by the fact that non-weaned lambs and kids are less likely to get exposed to diseases because they are managed carefully and do not often mix with adults’ animals during feeding time. Adults from the age of 6 months onwards also begin to develop immunity to certain parasite species, having been exposed to the environment for a while. As the longitudinal study was to be carried out over a period of 1 year, the maximum age of the animals to be chosen would also be considered to ensure that they would be in the growth period during the study period. Each animal selected within the target herd received a tag for monitoring. The farmers agreed to keep all selected animals for at least the duration of the experiment. Any animal among the selected group that was lost, died, or was sold was replaced by another animal of the same age group and species that had been previously identified and sampled during the baseline survey. To this end, three additional animals were identified in each herd to replace the initially selected animals if necessary. In cases where there were no animals left in reserve for replacement, the data were considered missing and removed from the database analyzed. For this purpose, one of inclusion criteria of farmers in this study was to own at least 10 small ruminants aged between 2 and 6 months.

Two veterinary technicians were hired to monitor the herds. These technicians, who are based in the municipality, visit the farmers at least once a week as part of their herd monitoring duties. During their weekly visits, the agents inspected the herd and the various areas of the farm, including housing, feed and medication storage areas, and farming equipment. They discussed with the farmers the various incidents that had occurred (cases of disease, deaths, missing animals, sales, thefts, etc.) since the last visit, as well as any solutions that had been implemented. All this information is recorded in the monitoring logbook provided for this purpose on each farm. This study was approved by the ILRI Ethics Committee following a detailed presentation of the various types of samples to be collected from the animals and the methods to be used.

### 2.3 Description of the intervention package

The intervention consisted of implementing our package in the Test herds while the Control herds continued their previous practices. Our package comprises the following components: (i) capacity building of the farmers, owners of Test herds, covering good management of small ruminant farming (health, nutrition, reproduction etc.); (ii) at least two rounds of deworming with levamisole were implemented in Test herds, unlike the Control herds, which were not dewormed during the period of the study; (iii) vaccination against PPR in the Test herds at the beginning of the intervention.

Two workshops were organized for livestock farmers on best practices:

- For small ruminant feeding, the topics discussed were as follows: (i) types of feed available in the local environment for small ruminants; (ii) different feed groups for small ruminants; (iii) how feed should be distributed among small ruminants;
- For small ruminant health, the topics discussed were as follows: (i) how to recognize a sick animal and the appropriate steps to take, including when to call the veterinarian; (ii) clinical signs of parasitic infections in animals and possible treatments; (iii) a prophylaxis plan;
- For small ruminant housing, the topics discussed were as follows: (i) importance and types of habitats; (ii) density; (iii) orientation; (iv) distance between types of infrastructure on the farm;
- For small ruminant farming hygiene the topics discussed were as follows: (i) hygiene of housing and equipment for small ruminants; (ii) biosecurity on small ruminant farms;
- For small ruminant reproduction, the topics discussed were as follows: (i) male-to-female ratio; (ii) male career management; (iii) female career management; (iv) selecting a good breeding animal.

Adoption of the package was monitored by staff recruited for this purpose. During their weekly visits, these staff members observed how the training provided for the farmers was being put into practice and brought any observed shortcomings to the farmers’ attention so that they could be corrected.

All eligible animals in the Test herds were dewormed with levamisole at the onset of the study. The dosage was adjusted according to each animal’s weight and age. This treatment was repeated during the mid-term visit to the Test herds.

All eligible animals were vaccinated against PPR at the start of the study in the Test herds and at the end of the study in the Control herds.

The program was therefore implemented in the Test herds for 1 year, starting at the beginning of the experiment. The Control herds received this program at the end of the experiment as compensation.

### 2.4 Sample size estimation

#### 2.4.1 Sample size for comparing two groups (outcome being animal level weight gain)

Our study was a Test vs. Control study in which small ruminants we enrolled (recruited at 2–6 months of age) and monitored for liveweight gain for 12 months. We collected data on growth performance (average weight gain per group, in grams/day) in 4-week intervals. Using weight gain as a key productivity indicator, the required sample size (Equation 1) for comparing herds that applied the package (group 1) versus herds that did not apply the package (group 2) was computed from the equation of (11):


$$n=2\frac{\left[{\left(Z_{\alpha }+Z_{\beta }\right)}^{2}\sigma^{2}\right]}{{\left(\mu_{1}-\mu_{2}\right)}^{2}}$$ (1)

where *n* = is the required sample size per group; *Z* = is the value of *Z* required for a 95% confidence (1.96); *Z_β_* = is the value of *Z* required for the power of the study of 80%; $\sigma^{2}$ = is the estimated common variance in the two groups (280), computed from previous studies (1, 12–14) and *μ*_1_ − *μ*_2_ = is the estimated difference in the mean Average Daily Weight Gain of the two groups (22 g/animal/day). The computed sample size was 10 herds for Test and Control group.

#### 2.4.2 Sample size for comparing two groups (outcome being parasite prevalence)

The prevalence of GIPs in small ruminants was estimated at 60% (15–17). An appropriate combination of several anti-helminthics can achieve a deworming efficacy of up to 90% (18). We therefore expected a reduction of 54% in prevalence following an intervention, which represent a 90% reduction in the estimated prevalence. So, the residual prevalence would be 6% after good deworming and hygiene practices on farms through application of the herd health package.

The required sample size (Equation 2) for 80% power and a statistical significance of 95% is derived from the two-sample binomial comparison equation (11):


$$n=\frac{{\left(Z_{\frac{\alpha }{2}}+Z_{\beta }\right)}^{2}(p_{0}(1-p_{0})+p_{1}(1-p_{1}))}{d^{2}}$$ (2)

Where *Z*_*α*/2_ is the standard normal deviation for *α* = 1.960; *Z_β_* is the standard normal deviation for *β* = 0.84 *p*_0_ = 60%, *p*_1_ = 6%, and the estimated effect size, *d* = 54%. Using this equation, *n* = 8. Thus, we require eight herds per group for the gastrointestinal assessment at a single timepoint. This calculation is for estimating the prevalence at each of the timepoints measured.

The sample size for the average daily weight gain comparison is larger than the sample size of the gastrointestinal assessment, and considering that we are doing the two activities in the same herds and villages, we decided to use the largest sample size, which was10 herds per group. We have increased the number of herds from 10 to 12 in anticipation of cases of withdrawal from the study.

In each Test and Control group, we decided to sample 5 animals per herd to have 10 animals per village, so we sampled a total of 120 animals for this study. In addition, we decided to select 3 animals from each herd to serve as a reserve in case of death, loss, or sale. This increased the likelihood that we would have 120 animals available for the entire duration of the experiment. In this study, we compared animals from the experimental herds with those from the Control herds, both at the beginning and at the end of the study. We assessed the extent to which integrated herd management could reduce GIP infection rates and improve productivity.

### 2.5 Fecal sample collection for parasite detection and identification

Parasites were identified using coprology techniques. This technique involved sampling fresh feces from small ruminants.

A sample of around 10 g of feces was taken directly from the rectum of the animal, well restrained, in a plastic bag. We added 3–5 drops of formaldehyde diluted to 10% for good conservation of any parasite eggs contained in the feces (19, 20). The samples were kept in an ice box at 4 °C for the duration of the fieldwork, before being transported to the Central Veterinary Laboratory in Bamako, where they were processed.

At the laboratory, two methods were sequentially used for coprology. A first method was the flotation technique to assess the presence/absence of parasite eggs and to determine their genera (21–23). The parasitic load in sampled animals was estimated using the modified McMaster method (24) by determining the number of eggs per gram (EPG) in the feces.

### 2.6 Laboratory analysis methods

The coproscopic analysis was performed using a flotation technique followed by a quantitative assessment with the modified McMaster method.

For the flotation technique, 5–10 g of feces were mashed in a beaker with 20–30 mL of saline solution. The mixture was filtered through a double gauze or sieve into a first tube, and the filtrate was then poured into a second tube until a meniscus formed. A coverslip was placed on the meniscus and left undisturbed for 10–15 min. The coverslip was then carefully removed, placed on a microscope slide, and examined under a microscope using the 10× objective.

Samples that tested positive by flotation were subsequently analyzed using the modified McMaster method to determine parasite load. In this procedure, 5 g of feces were dissolved in 60 mL of saturated NaCl solution with a concentration of 40% and a density of 1.20. The mixture was filtered through double gauze into a beaker and homogenized by shaking. A small volume of the filtrate was collected using a bulb pipette and used to fill both chambers of a Paracytometer slide. Each slide chamber volume is 0.3 mL. After allowing 5–10 min for the eggs to float and adhere to the coverslip, the slide was examined under a microscope using the 10× objective. All eggs were counted by parasite type within the marked grids of the two chambers, and the number of eggs counted was used to calculate the number of eggs per gram of feces (EPG) using the following formula (Equation 3):


$$\mathrm{EPG}=\frac{n\times V}{P\times v}$$ (3)

Where EPG is the number of eggs per gram of feces; *n* is the total number of eggs counted in the two examined cells; *V* is the total volume of fecal suspension, i.e., 60 mL; *P* is the weight of feces used, i.e., 5 g and *v* is total volume examined in the two cells of the McMaster slide, i.e., 0.60 mL (0.30 mL per cell).

### 2.7 Survey questionnaire

After fecal samples were taken from the animals, a questionnaire was administered to the farmers. The survey was administered face-to-face to the farmer by the researcher, assisted by a translator recruited locally and trained on the questionnaire. This questionnaire was administered to the same farmer at the baseline and endline of the study. The questionnaire collected information on the household, livestock, type of animal housing, animal feed, diseases, medical and sanitary prophylaxis, health care, reproduction, household resources, and income. All these data were used to identify risk factors for gastrointestinal parasitosis.

The questionnaire was validated by animal health professionals working in the study area. A consensus was reached on the translation of various technical terms based on the local language. The translator recruited is also a health professional and a key stakeholder in the Farakala Innovation Platform, an association that brings together all stakeholders in the municipality’s small ruminant livestock sector.

### 2.8 Statistical analysis

We calculated the prevalence of each genus of parasite among the sampled animals. We consider a result positive for parasites if an animal has at least one egg of the parasite in its feces and negative when there is no egg of this parasite. The prevalence was calculated according to the following formula (Equation 4):


$$P=\frac{\mathrm{NP}}{\mathrm{NT}}*100\;$$ (4)

Where *P* is the prevalence of the parasite; *NP* is the number of positive animals for the parasite; and *NT* is the number of tested animals.

We calculated the incidence of each type of parasite among the sampled animals by summing the number of animals tested positive for that parasite in each herd. The incidence therefore represents the total number of animals that tested positive for the target parasite. We tested the normality of continuous dependent variables and checked their relationship with independent variables accordingly with ANOVA Test, Kruskal Test, or Linear regressions. Since we have more than 100 records, we used the Kolmogorov–Smirnov Test instead of the Shapiro–Wilk Test to test the normality of data.

Using various models, including Linear regression models, generalized linear models (GLMs), generalized linear mixed-effects models (GLMMs), and negative binomial mixed models (GlmmTMBs) according to the type and nature of the variables (Table 1), we assessed the effect of the package on animals in the Test herds that implemented it, compared to those in the Control herds.

**Table 1:** List of outcomes and the type of models used for a statistical analysis.

Outcomes	Type of data	Models used for analysis
Effect of package implementation on parasite incidence	Numerical (count of positive animals)	Generalized linear models (GLMs)
Effect of package implementation on parasite prevalence	Numerical (proportion of positive animals among tested)	Generalized linear mixed-effects models (GLMMs)
Effect of package implementation on average daily weight	Numerical (average daily weight gain)	Linear regression models
Effect of package implementation on parasite load	Numerical (parasite eggs per gram of feces)	Negative binomial mixed model (GlmmTMBs)
Effect of package implementation on herd size evolution	Numerical (herd size)	Generalized linear models (GLMs)

We used the long format of binomial GLMM and GlmmTMB, with an interaction between group and period (group*period). This interaction acts exactly like a baseline-adjusted analysis to handle baseline imbalance.

Statistical analyses were carried out using R software (version R 4.4.1) and various packages, including the following: readxl; tidyverse; lme4; ggplot2; emmeans; readr; dplyr with following functions: readxl::read_xlsx; summary; ks.test; kruskal.test; lm; confint.default; aov; boxplot; glm; exp.; ggplot; read_tsv; glmer; glmmTMB.

The study area map was made with QGIS (version 3.34.10).

## 3 Results

### 3.1 Health issues reported by farmers for the whole period of the intervention in the visited herds

**Figure 2:**
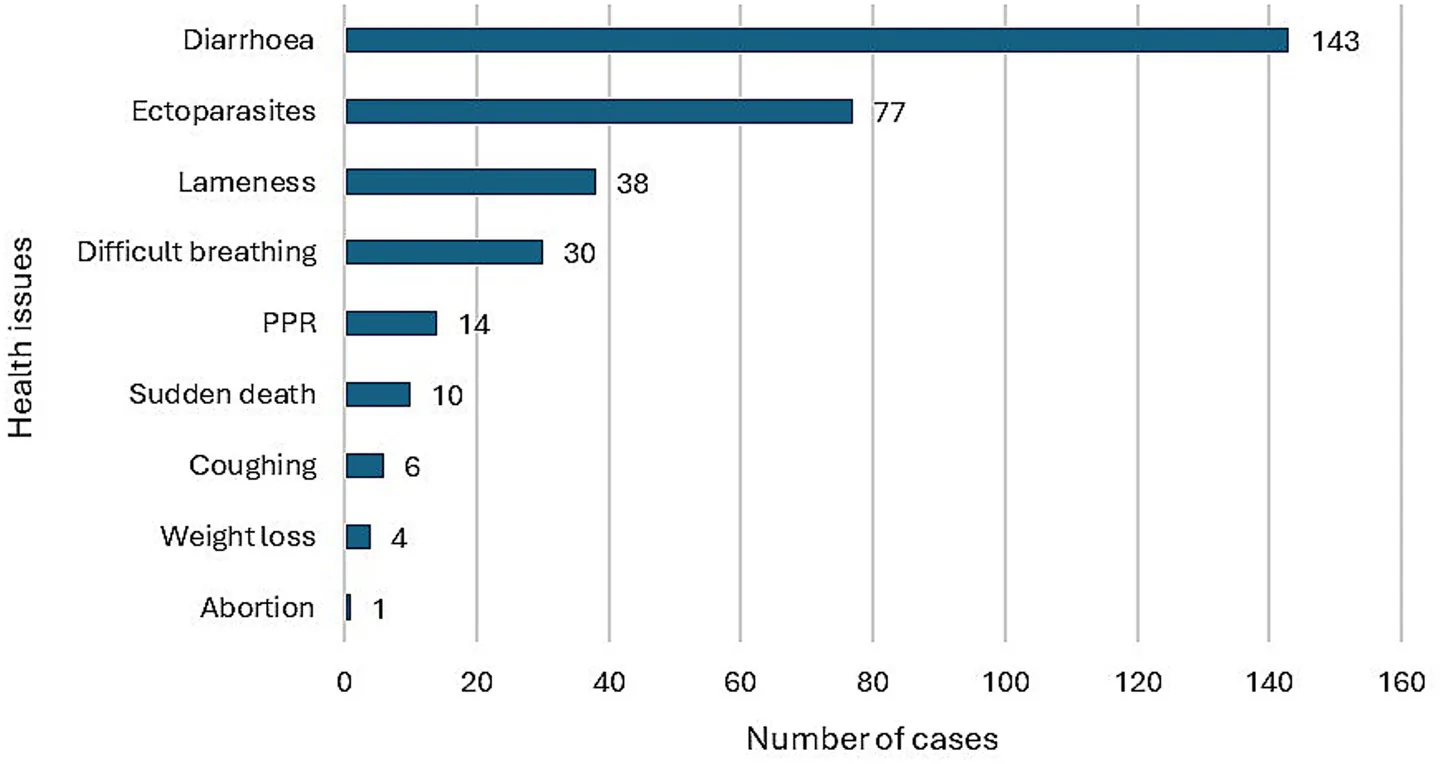
Histogram of small ruminant clinical health issues reported by farmers during the period of the intervention. PPR stands for Peste des Petits Ruminants.

Diarrhea was the most reported health problem by the farmers in all herds visited at the end of the intervention with (143 cases), followed by ectoparasitic infestation (77 cases), and lameness (38 cases). The least reported health issue was abortion (1 case) (Figure 2).

A logistic regression showed that there were significantly (*p* < 0.05) fewer health problems in Test herds (OR: 0.62) than in Control herds (Table 2).

**Table 2:** Influence of the implementation of the herd health package on the frequency (number of cases for 1 year) of health issues evaluated by a GLM model.

Variables	Odds ratio (OR)	Confidence intervals (95%)	Log-Odds Standard Error	*z* value	Pr(>|*z*|)
(Herd’s Intercept)	16.67	[14.46, 19.08]	0.07	39.79	***
Health issues in Test herd	0.62	[0.49, 0.77]	0.11	−4.24	***

Significance codes: 0 ‘***’ 0.001 ‘**’ 0.01 ‘*’ 0.05 ‘.’ 0.1 ‘ ’ 1.

### 3.2 Results of coprology

By comparing the number of infected animals between the beginning and end of the intervention and also the parasite load, we noticed a significant decrease in the herds that applied our package (Table 3).

**Table 3:** Impact of herd health package on animal infection and parasite load.

Herd type	Control		Treatment		All herds	
Period	Before intervention	After intervention	Before intervention	After intervention	Before intervention	After intervention
Animals infected by at least one species of parasites	41	21	43	9	84	30
Eggs of Strongylid nematodes	3,552	1,280	8,688	240	12,240	1,520
Eggs of *Moniezia* spp.	10,520	0	2060	320	12,580	320
Oocysts of *Eimeria* spp.	1,620	820	1,300	60	2,920	880
Eggs of *Trichuris* spp.	20	60	180	0	200	60
Total parasites load	15,712	2,160	12,228	620	27,940	2,780
Total animals tested	60	60	60	60	120	120

At the baseline, four genera of GIP eggs were found by the coprological tests: Strongylid nematodes were the most prevalent (51.70%), followed by *Eimeria* spp. (30%), *Moniezia* spp. (22.50%), and *Trichuris* spp., which was the least prevalent (5.80%). In sheep, Strongylid nematodes (54.88%) were the most prevalent parasite genus, whereas *Trichuris* spp. (7.32%) were the least prevalent. In goats, Strongylid nematodes (44.74%) and *Eimeria* spp. (44.74%) were the most prevalent genera, and *Trichuris* spp. (2.63%) was the least prevalent parasite genus identified (Table 4).

**Table 4:** Prevalence of gastrointestinal parasites in the tested animals according to species.

Parasites	Goat			Sheep		
	Before intervention	After intervention	Total tested	Before intervention	After intervention	Total tested
Strongylid nematodes	44.74% (*n* = 17)	23.68% (*n* = 09)	38	54.88% (*n* = 45)	14.63% (*n* = 12)	82
*Moniezia* spp.	13.16% (*n* = 05)	0% (*n* = 00)	38	26.83% (*n* = 22)	2.44% (*n* = 02)	82
*Eimeria* spp.	44.74% (*n* = 17)	18.42% (*n* = 07)	38	23.17% (*n* = 19)	13.41% (*n* = 11)	82
*Trichuris* spp.	2.63% (*n* = 01)	0% (*n* = 00)	38	7.32% (*n* = 06)	3.66% (*n* = 03)	82

At the endline, four genera of GIP eggs were found by the coprological tests: Strongylid nematodes were the most prevalent (17.50%), followed by *Eimeria* spp. (15%), *Trichuris* spp., (2.50%), and *Moniezia* spp., which was the least prevalent (1.67%). In sheep, Strongylid nematodes (14.63%) were the most prevalent parasite genus, whereas *Moniezia* spp. (2.44%) was the least prevalent. In goats, Strongylid nematodes (23.68%) were the most prevalent genus, and *Moniezia* spp. and *Trichuris* spp. (0%) were the least prevalent parasite genera identified (Table 4).

**Figure 3:**
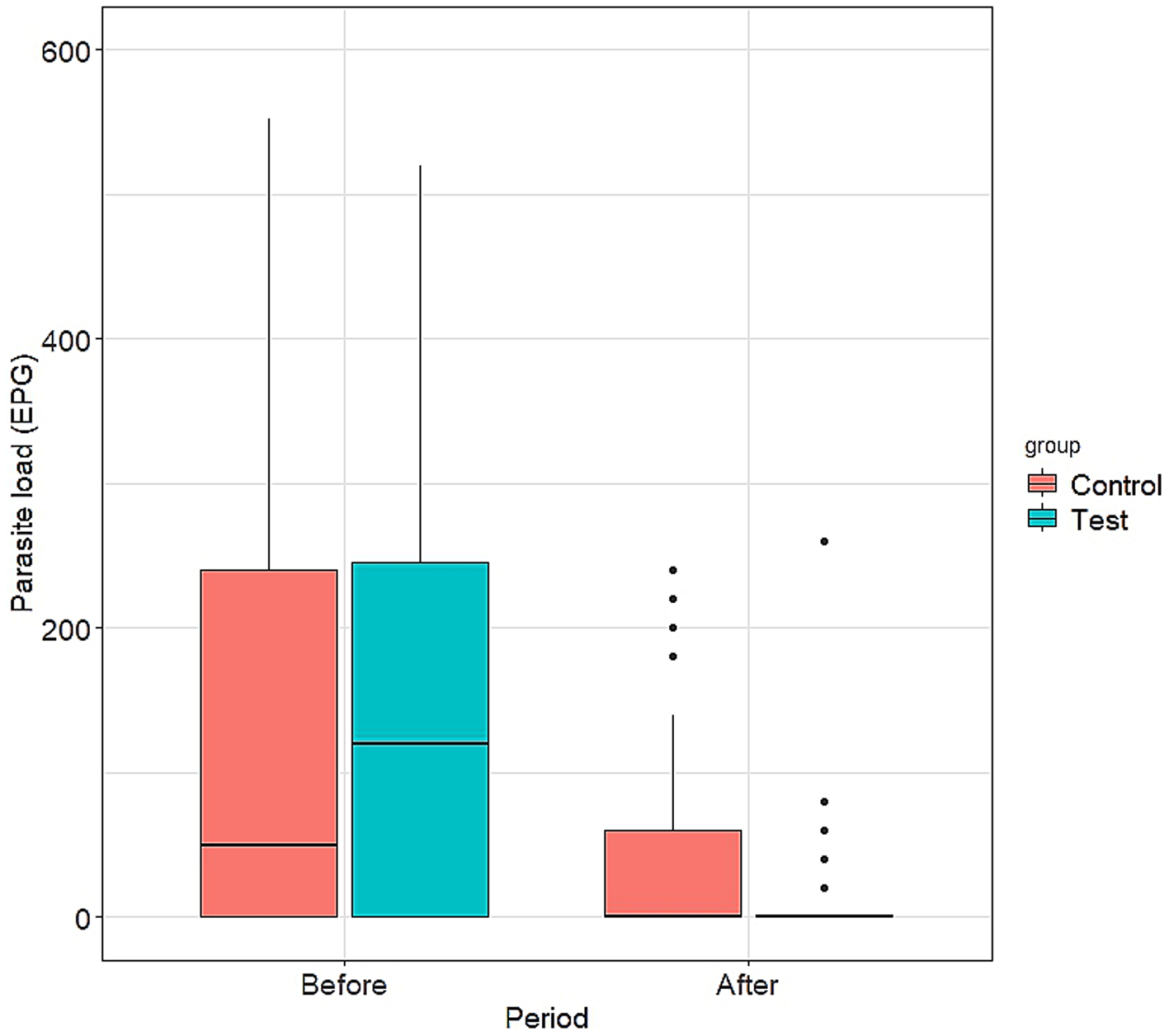
Distribution (boxplot) of small ruminants’ gastrointestinal parasite load according to herd types (control/test) and period (before/after) of the intervention. EPG stands for eggs per gram.

The logistic regression indicated that, at the baseline, sheep were more heavily infested than goats (OR = 2.14), and Test herds were less heavily infested than Control herds (OR = 0.78) (Table 5, Figure 3).

**Table 5:** Level of infection of small ruminants by gastrointestinal parasites according to their species and the type of herd evaluated by a GLM model.

Variables	Odds ratio (OR)		Confidence intervals (95%)		Log-Odds Standard Error		*z* value		Pr(>|*z*|)
	Before	After	Before	After	Before	After	Before	After	
(Species’ Intercept)	130.84	17.37	[127.24, 134.51]	[16.08, 18.73]	0.01	0.04	343.68	73.34	***
Sheep	2.14	1.49	[2.08, 2.21]	[1.36, 1.63]	0.02	0.04	48.66	8.92	***
(Herd’s Intercept)	261.87	36.00	[257.79, 265.99]	[34.50, 37.54]	0.01	0.02	697.96	166.55	***
Test Herd	0.78	0.30	[0.76, 0.80]	[0.26, 0.31]	0.01	0.05	−20.79	−27.39	***

Significance codes: 0 ‘***’ 0.001 ‘**’ 0.01 ‘*’ 0.05 ‘.’ 0.1 ‘ ’ 1.

Results of logistic regression showed at the endline the same trend that sheep are more heavily infested than goats (OR = 1.49), and Test herds are less heavily infested than Control herds (OR = 0.30) (Table 5’ Figure 3).

### 3.3 Effect of herd health package on herd size

The Kruskal–Wallis test on herd growth rates showed a significant difference between the medians of at least two groups (*p* < 0.05).

Linear regression showed a significant difference between the growth rates of the Test and Control herds (*p* < 0.05). The growth rate of the Test herds (*β* = 23.70%) was higher than that of the Control herds (Table 6).

**Table 6:** Influence of the implementation of the herd health package on the herd growth rate evaluated by a linear regression model.

Variables	Coefficients (%)	Confidence intervals (95%)	Log count Standard Error	*t* value	Pr(>|*t*|)
(Herd’s Intercept)	17.62	[7.77, 27.47]	4.98	3.54	***
Test Herd	23.70	[9.77, 37.63]	7.04	3.37	**
(Village’s Intercept)	16.97	[−1.81, 35.76]	9.48	1.79	.
Gniriwani	28.27	[1.70, 54.84]	13.40	2.11	*
Ifola	−27.86	[−54.43, −1.30]	13.40	−2.08	*
Kalifabougou	24.03	[−2.54, 50.59]	13.40	1.79	.
Mpedougou	43.99	[17.42, 70.56]	13.40	3.28	**
Notanso	79.46	[52.89, 106.02]	13.40	5.93	***

Significance codes: 0 ‘***’ 0.001 ‘**’ 0.01 ‘*’ 0.05 ‘.’ 0.1 ‘ ’ 1.

Linear regression also showed a significant difference between the growth rates of the village herds (*p* < 0.05). The villages of Notanso (*β* = 79.45%), Mpedougou (*β* = 43.99%), Gniriwani (*β* = 28.27%), and Kalifabougou (*β* = 24.03%) have the highest herd growth rates, while the village of Ifola (*β* = −27.86%) has the lowest herd growth rate (Table 6).

**Figure 4:**
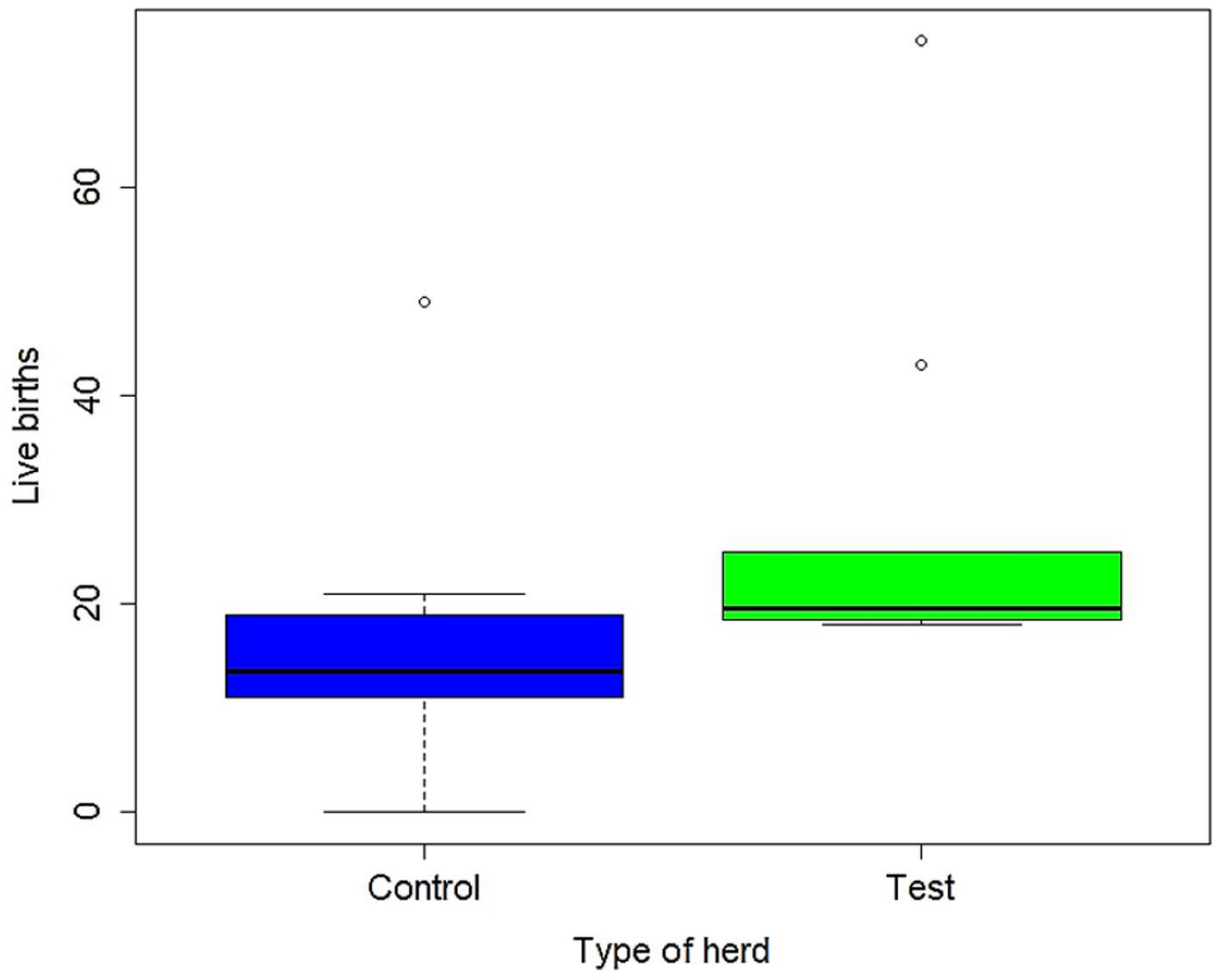
Distribution (boxplot) of small ruminants’ live births according to the type of herds.

A logistic regression showed that the number of small ruminant live births was significantly higher in the Test herds than in the Control herds (*p* < 0.05). The number of live births in the Test herds was 1.65 times higher than in the Control herds (Table 7, Figure 4).

**Table 7:** Influence of the location where the herd health package was implemented on the number of livebirths evaluated by a generalized linear regression model (GLM).

Variables	Odds ratio (OR)	Confidence intervals (95%)	Log-Odds Standard Error	*z* value	Pr(>|*z*|)
(Herd’s Intercept)	16.25	[15.25, 17.29]	0.03	87.06	***
Test Herd	1.65	[1.53, 1.79]	0.04	12.36	***
(Village’s Intercept)	17.50	[15.03, 20.22]	0.08	37.86	***
Ifola	1.91	[1.60, 2.30]	0.09	6.96	***
Nangola	1.23	[1.00, 1.50]	0.10	2.02	*
Wayere	2.49	[2.09, 2.97]	0.09	10.17	***

Significance codes: 0 ‘***’ 0.001 ‘**’ 0.01 ‘*’ 0.05 ‘.’ 0.1 ‘ ’ 1.

The logistic regression indicated that the number of live births was significantly higher in some villages (*p* < 0.05). The number of live births was significantly higher in the villages of Wayere (OR: 2.49), Ifola (OR: 1.91), and Nangola (OR: 1.23) (Table 7).

### 3.4 Effect of herd health package on average weight gain of small ruminants

**Figure 5:**
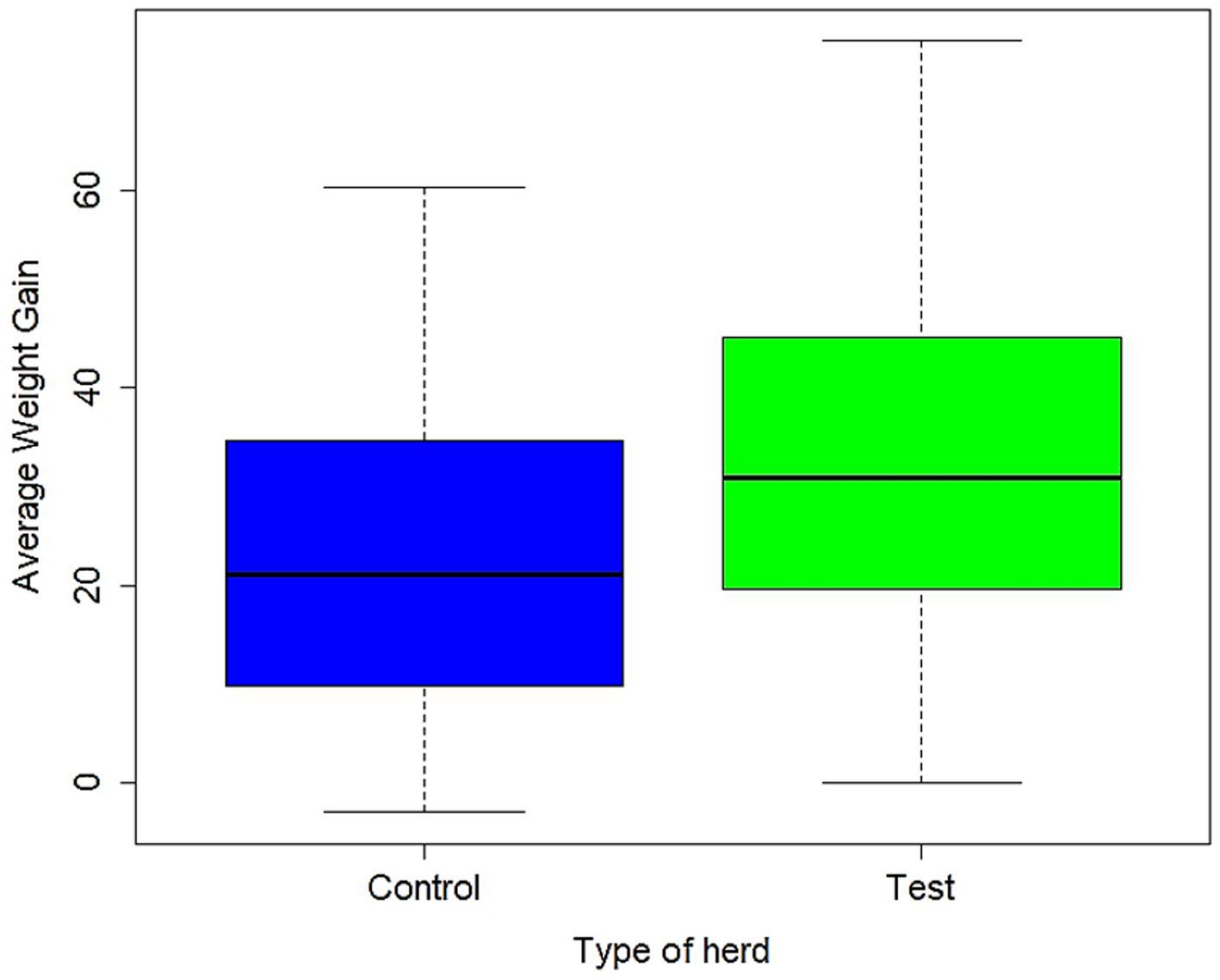
Distribution (boxplot) of small ruminants’ average weight gain according to the type of herds.

A linear regression showed a significant difference between the average weight gain of the Test and Control herds (*p* < 0.05). The average weight gain of the Test herds (*β* = 08.80 g/day) was higher than that of the Control herds (Table 8, Figure 5).

**Table 8:** Influence of the implementation of the herd health package on the average weight gain of small ruminants evaluated by a linear regression model.

Variables	Coefficients (g/day)	Confidence intervals (95%)	Log count Standard Error	*t* value	Pr(>|*t*|)
(Herd’s Intercept)	23.16	[19.10, 27.22]	2.05	11.28	***
Test Herd	8.80	[3.05, 14.55]	2.90	3.03	**
(Village’s Intercept)	25.15	[15.82, 34.48]	4.71	5.35	***
Ifola	14.16	[0.97, 27.35]	6.65	2.13	*
Wayere	20.35	[7.15, 33.54]	6.65	3.06	**

Significance codes: 0 ‘***’ 0.001 ‘**’ 0.01 ‘*’ 0.05 ‘.’ 0.1 ‘ ’ 1.

The linear regression also showed a significant difference between the average weight gain of some village herds (*p* < 0.05). The villages of Wayere (*β* = 20.35 g/day) and Ifola (*β* = 14.16 g/day) had the highest average weight gain (Table 8).

### 3.5 Effect of herd health on gastrointestinal parasite incidence

**Figure 6:**
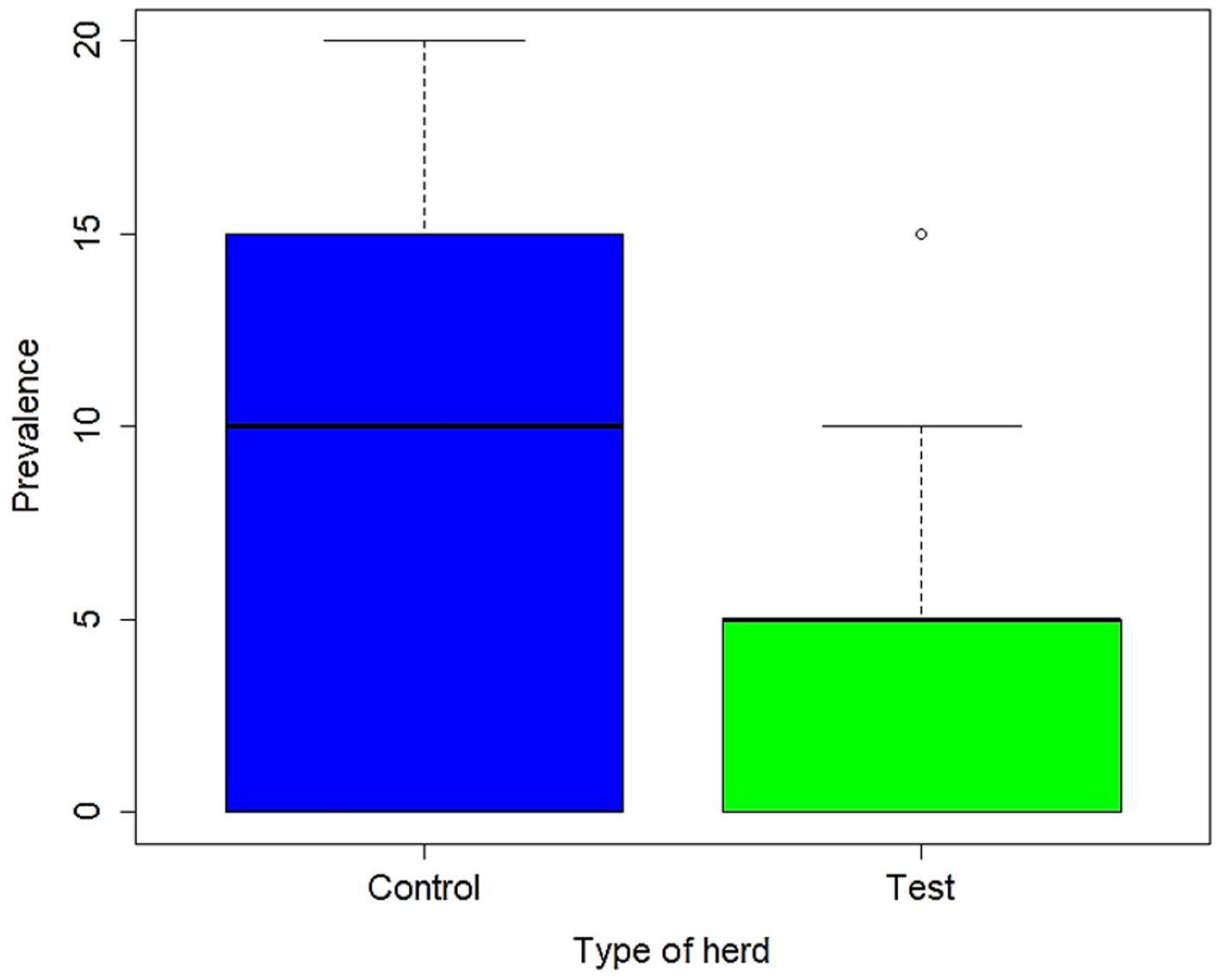
Distribution (boxplot) of prevalence of small ruminants’ gastrointestinal parasites according to herd types. GIPs stands for gastrointestinal parasites.

The logistic regression showed that there is a significant difference in GIP incidence between Test and Control herds (*p* < 0.05). The incidence of GIP is significantly less important in Test herds (OR: 0.43) than in Control herds (Tables 3, 9; Figure 6).

**Table 9:** Influence of the implementation of the herd health package on incidence of gastrointestinal parasites evaluated by a generalized linear regression model (GLM).

Variables	Odds ratio (OR)	Confidence intervals (95%)	Log-Odds Standard Error	*z* value	Pr(>|*z*|)
(Herd’s Intercept)	0.35	[0.22, 0.52]	0.22	−4.81	***
Test Herd	0.43	[0.19, 0.91]	0.40	−2.13	*

Significance codes: 0 ‘***’ 0.001 ‘**’ 0.01 ‘*’ 0.05 ‘.’ 0.1 ‘ ’ 1.

### 3.6 Impact of the intervention on the prevalence of GIP

The binomial GLMM model, including a random herd effect, indicated a significant effect of the intervention (group × period interaction: OR = 1.06, *p* > 0.05).

The Test and Control groups were not different before the intervention (*p* > 0.05).

These results show that the intervention led to a real and significant reduction in the prevalence of the infection (Table 10).

**Table 10:** Impact of the intervention on the prevalence of gastrointestinal parasites evaluated by a generalized linear mixed-effects model (GLMM).

Variables	Odds ratio (OR)	Confidence intervals (95%)	Log Odds Standard Error	*z* value	Pr(>|*z*|)
(Intercept)	0.16	[0.11, 0.22]	0.17	−10.9	***
groupTest	1.06	[0.67, 1.68]	0.24	0.2	
periodafter	0.48	[0.27, 0.83]	0.28	−2.6	**
groupTest:periodafter	0.39	[0.15, 0.98]	0.47	−2.0	*

Significance codes: 0 ‘***’ 0.001 ‘**’ 0.01 ‘*’ 0.05 ‘.’ 0.1 ‘ ’ 1.

*(Intercept):* The baseline odds of an animal testing positive in the Control group during the before.

*period.groupTest:* The odds ratio comparing the Test group to the Control group at baseline (before). A value significantly different from 1 mathematically confirms and measures your baseline imbalance.

*periodafter:* The odds ratio of an animal testing positive in the Control group changing from before to after. It captures natural background change over time.

*groupTest:periodafter:* This is your critical treatment effect. It is the Ratio of Odds Ratios (ROR). It tests if the trajectory of the Test group from before to after significantly diverges from the trajectory of the Control group, completely accounting for that initial baseline imbalance.

### 3.7 Impact of the intervention on parasite load in animals

The negative binomial model, including a random herd effect, indicated a non-significant effect for the intervention, where group × period interaction: incidence rate ratio (IRR) = 3.22, *p* > 0.05.

The Test and Control groups were different before the intervention (*p* < 0.05).

These results show that the intervention does not lead to a real and significant difference in the reduction of GIP load between the Control and Test herds (Table 11).

**Table 11:** Impact of the intervention on parasite load in animals evaluated by a negative binomial mixed model (GlmmTMB).

Variables	Incidence rate ratio (IRR)	Confidence intervals (95%)	Log count Standard Error	*z* value	Pr(>|*z*|)
(Intercept)	34.38	[15.47, 76.39]	0.41	8.68	***
groupTest	0.25	[0.07, 0.90]	0.65	−2.13	*
periodBefore	7.35	[2.56, 21.09]	0.54	3.71	***
groupTest:periodBefore	3.22	[0.63, 16.45]	0.83	1.40	

Significance codes: 0 ‘***’ 0.001 ‘**’ 0.01 ‘*’ 0.05 ‘.’ 0.1 ‘ ’ 1.

*(Intercept):* The baseline event rate for the reference group during the reference.

*groupTest* (Main Effect): The incidence rate ratio (IRR) comparing your treatment group to the reference group specifically during the baseline period (when period = 0).

*periodBefore* (Main Effect): The IRR tracking the change over time specifically for the reference group (when group = 0).

*groupTest:periodBefore* (Interaction Term): This is a Ratio of IRRs. It tells how much the effect of the group changes over time.

## 4 Discussion

### 4.1 Farms that applied the herd health package reported fewer health problems

Diarrhea was the most reported health issue in the monitored herds. Diarrhea is a common symptom of several small ruminant diseases, particularly GIPs, which are the most frequent pathologies in small ruminant farming. Gastrointestinal nematode infections represent an important challenge for small ruminant farmers in Sub-Saharan Africa (25). Herds that applied the herd health package reported fewer health problems. This could be due to the efficacy of the interventions, which included vaccination against PPR, deworming, and improvement of habitat and biosecurity. These interventions could have reduced the risk of disease occurrence and spread.

### 4.2 Farms that applied the herd health package showed an increase in herd size

The implementation of the herd health package in the Test herds significantly increased their herd size compared to the Control herds, which did not implement the package. Similar results were found by Moliso et al. (10) in Ethiopia, who reported that herd health packages have a profoundly positive effect on small ruminant herd size, primarily by increasing birth rates and significantly reducing mortality. These benefits result from a comprehensive, proactive strategy that addresses the major obstacles to small ruminant productivity, such as disease, nutrition, and management inefficiencies. Studies in regions like West Africa and Ethiopia have documented that implementing these packages can double flock sizes within a single year (26).

### 4.3 Herds that applied the health package showed higher average weight gain

Animals in Test herds showed a higher weight gain than those in Control herds. Vaccination against PPR can reduce the morbidity and increase the weight gain in small ruminants (4). In a study conducted by Korir in 2008 the overnight housing, deworming and supplementary feeding had a significant effect on the average weight gain on weaner goats (27). Sharma in 2014 and Bessel in 2018 also showed that deworming has an positive effect on average weight gain of small ruminants (28, 29). All these components taken separately have a positive effect on the average daily gain. The simultaneous implementation of these actions in the herd health package will potentiate their effects. The animals used in this study were between 2 and 6 months old. It should be noted that after 6 months of age, small ruminants experience a natural decline in their growth rate, which may be a confounding factor that we did not consider in this study.

### 4.4 Farms that applied the herd health package showed reduced parasite incidence and prevalence

The incidence and prevalence of GIPs in Control herds was significantly higher than in the Test herds, revealing the efficacy of regular deworming in Test herds. At least two cases of deworming with levamisole were implemented in Test herds unlike the Control herds, which did not deworm during the period of the study. Parasites thrive when animals are poorly managed, stressed, or undernourished, and a comprehensive health plan can break their lifecycle, enhance animal immunity, and reduce the reliance on chemical treatments. Effective herd health programs significantly reduce gastrointestinal parasite incidence and prevalence in small ruminants by improving overall health, managing factors like nutrition and stress, and implementing strategic parasite control measures. This leads to lower morbidity and mortality rates, as well as increased productivity. Key herd management practices include improved hygiene, proper nutrition, strategic deworming that incorporates concepts like preserving a “refugia,” and separating young animals from adults to decrease infection pressure (30).

Our study showed a significant decrease in the incidence and prevalence of GIPs in herds that implemented the integrated package. At the same time, we also observed a decrease in parasite load/intensity, but the difference was not significant between the herds in the Test group and those in the Control group. At the start of the study, we selected young animals, which had reached adulthood by the end after 1 year of follow-up. Adult small ruminants exposed to GIPs over a long period may develop natural protective immunity against these parasites. Even if they do not eliminate all GIP, either through an appropriate deworming program or naturally, this immunity can help reduce the parasites’ ability to reproduce, thereby decreasing the number of eggs they can excrete into pastures. Young small ruminants lack this immunity and can become heavily infested and highly vulnerable (31). This can be a hypothesis to explain the same level of parasite loads in the test and control groups 1 year later. It should be noted, however, that the lack of immunological data to support this hypothesis limits the validity of this explanation.

Study design and small sample size are important limitations. This could have led to an overestimation of the effect. We suggest that future research reconfirm these findings by conducting larger-scale studies.

Another limitation of this study is that the health problems identified in the herds are based on clinical signs, with the exception of GIPs, which were identified through laboratory test results. For example, cases of diarrhea are not exclusively caused by parasites and may therefore be of viral or bacterial origin.

The study used levamisole for deworming. Resistance to anthelmintics is a growing concern in West Africa. It would have been interesting to evaluate the efficacy of this antiparasitic drug using the Fecal Egg Count Reduction Test (FECRT) by measuring changes in the number of parasite eggs after treatment. This constitutes a limitation of this study.

## 5 Conclusion

Raising small ruminants is an income-generating activity that is not too demanding and is often recommended for low-income rural households. When practiced rigorously, this type of farming produces very good results. The study shows that the herd health package (regular targeted deworming, vaccination against major diseases, and improved feeding and housing) has led to measurable performance gains: a significant increase in average daily weight gain and a notable reduction in incidence and prevalence of GIPs. It would be good if this study could be conducted with a larger sample size and across a wider geographic area. However, in view of these results, it is recommended that this approach be extended to other similar farms, combining training for farmers and epidemiological monitoring to maximize the impact and limit risks, particularly anthelmintic resistance and the inappropriate use of veterinary medicines.

## Data Availability

The raw data supporting the conclusions of this article will be made available by the authors, without undue reservation.
